# LAMBDA: A Prophage Detection Benchmark for Genomic Language Models

**DOI:** 10.64898/2026.03.26.714501

**Published:** 2026-03-26

**Authors:** LeAnn M. Lindsey, Nicole L. Pershing, Keith Dufault-Thompson, Ho-jin Gwak, Anisa Habib, Aaron Schindler, Arjun Rakheja, June Round, W. Zac Stephens, Anne J. Blaschke, Hari Sundar, Xiaofang Jiang

**Affiliations:** 1 National Library of Medicine, National Institutes of Health, Bethesda, MD, USA; 2 Kalherts School of Computing, University of Utah, Salt Lake City, UT, USA; 3 Department of Pediatrics,School of Medicine, University of Utah, Salt Lake City, UT, USA; 4 Department of Pathology,School of Medicine, University of Utah, Salt Lake City, UT, USA; 5 Department of Computer Science, Tufts University, Medford, MA, USA; 6 Division of Computer Engineering, Hankuk University of Foreign Studies, Yongin, Republic of Korea

**Keywords:** DNA language model, bacteriophage, prophage, genomic benchmark

## Abstract

Transformer-based genomic sequence models represent an emerging frontier in computational biology. Yet, their embeddings have not yet shown the same level of predictive power as natural and protein language models, indicating a gap between current implementations and theoretical promise. Existing benchmarks for DNA language models primarily focus on classifying regulatory elements in eukaryotic genomes, leaving open the fundamental question of whether these models learn sequence-level features across whole genomes. We introduce LAMBDA, a benchmark designed to rigorously evaluate genome language model embeddings through phage-bacteria sequence discrimination across four categories of increasing complexity: probing tasks, fine-tuning assessments, diagnostic tests, and genome-wide prophage detection. Our comprehensive analysis of current genomic language models provides novel insights into the importance of training data quality relative to model size, the need for domain-specific training, and the application of genomic language models for detecting prophage sequences. This benchmark represents a challenging genomic annotation task in the bacterial domain and addresses a key computational problem with direct relevance to microbiology and medicine.

## Background

Standardized benchmarks have been central to progress in the field of natural language processing. These benchmarks provide common frameworks for measuring performance, identify model strengths and weaknesses, and guide the development of increasingly capable models (1–3). Early benchmarks for genomic language models (gLMs) focused primarily on identifying short regulatory sequences in eukaryotic species, such as promoters (4), transcription factors (5, 6), and epigenetic marks (7). Two long-range benchmarks have recently been released (8, 9), which extend benchmark evaluations to longer sequence contexts, but remain focused on human regulatory genomics. While these benchmarks assess gLMs’ abilities to detect specific motifs and regulatory elements, they do not rigorously evaluate whether models can learn to identify functional boundaries or distinguish sequence types based on intrinsic properties, tasks that traditional methods accomplish through homology searches and explicit compositional features such as k-mer frequency distributions, GC content, and codon usage. Assessing whether gLMs can implicitly learn such patterns from sequence data alone would demonstrate a foundational understanding of DNA sequences with broad applicability in diverse genomic tasks.

In addition, several recent studies have questioned the “foundational” understanding of current genomic language models, demonstrating that, on current benchmarks, gLMs do not outperform randomly initialized models or simple supervised models for most genomic tasks (10, 11). These views stand in contrast to those of the model authors who claim that gLMs learn biologically meaningful representations of sequences, pointing to zero-shot performance across diverse tasks and the unsupervised discovery of genomic motifs as evidence of a foundational understanding (6, 7, 12–14).

Bacteriophages offer a compelling system in which to investigate these claims. Unlike model organisms with well-annotated genomes, the vast majority of phage genomes remain poorly characterized (15, 16). This makes them an ideal domain in which to test whether gLMs can identify meaningful sequence patterns, beyond what homology alone can reveal, a task with direct implications for the discovery of novel prophages.

Identifying the presence and location of prophages in host bacterial genomes is a significant goal for the microbiology and medical communities, but it remains a challenge (17). Phage genomes are extremely diverse, with closely related phage genomes often having a ‘mosaic’ nature (18–20), where homologous regions are separated by divergent or unrelated regions. Additionally, horizontal gene transfer in bacteria is commonly mediated by phages, which can lead to sections of a bacterial genome being integrated into the phage genome and then carried to different hosts. This process contributes to bacterial and phage diversity (21–23), but complicates the task of differentiating prophage and bacterial DNA. Lastly, phage genomes evolve rapidly, with high mutation rates and frequent gene loss, resulting in degraded prophage sequences being found throughout bacterial genomes (24). These factors result in a noisy genomic landscape in which distinguishing phage from bacterial sequences is complex.

The most accurate computational prophage detection tools rely on sequence homology with known phage genes (25–29). Phage gene databases have grown in size and scope, but remain biased toward phages that infect common human pathogens, making it challenging for these reference-based methods to detect novel prophages, especially in less well-studied bacterial species (30). With advances in machine learning, sequence-based approaches that are not dependent on homology searches have become more common (31, 32). These approaches rely on feature engineering to distinguish between the two classes, using gene density, average gene length, differences in GC content, strand switching frequency, and percentages of specific start codons (33). These are often combined with searches for genes annotated with the term “phage” (34), meaning that many of these methods still rely on sequence homology through gene annotation. One method employs a hybrid approach that integrates both homology features and sequence-based features into a deep learning classifier (35).

The LAMBDA benchmark aims to evaluate foundational genomic understanding in DNA language models using bacteria and bacteriophage sequences as a model system. Our benchmark quantifies the impact of pretraining using probing experiments on pretrained embeddings compared to embeddings from randomly initialized models. It systematically investigates sources of prediction error, including compositional bias, false positive rates, and sensitivity across functional categories. The benchmark also includes genome-wide prophage detection tests against gold-standard datasets to critically assess whether the reported prophage-detection capabilities of gLM models reflect genuine biological signal and generalize outside of a limited set of well-studied bacterial hosts. In addition to providing a challenging and novel task for gLMs, prophage identification in bacterial genomes is of significant biological interest, with implications for the spread of antibiotic resistance and microbial evolution.

## Results

### Evaluating the Power of Pretrained Sequence Representations.

The first phase of the LAMBDA benchmark is an evaluation of the power of the pretrained sequence representations of each model against a binary classification task, identifying a DNA sequence fragment as either phage or bacteria. This task is similar to the real-world task of metagenomic fragment annotation and was set up with a test set containing a 1:1 ratio of bacterial to phage sequences. To ensure that the bacterial sequences selected for the dataset did not include phage sequences, known phage sequences were identified using the Basic Local Alignment Search Tool (BLAST) (38) and removed from the dataset before data selection. To avoid data leakage arising from sequence similarity, clusters were split at the cluster level so that all segments from a given cluster appeared in only one partition (train, validation, or test).

For each model, pretrained embeddings were extracted and used as fixed representations to train two simple classifiers: a linear probe and a three-layer neural network classifier. The linear probe classifier is a single linear layer that maps the embeddings directly to the class predictions, testing whether the information is linearly accessible in the embedding representation. The three-layer neural network classifier is a small feed-forward network with two hidden layers and non-linear activations that is capable of measuring more complex, nonlinear structure in the embeddings. We compare this with the same tests using embeddings extracted from the same models with randomly initialized weights. The metric used in this evaluation is the Matthews Correlation Coefficient (MCC) because it provides a balanced measure derived from all four entries of the confusion matrix and is robust to class imbalance. The model produces a score between +1 and −1, with a value of zero reflecting random chance. For each classifier, we report the difference in MCC score, which quantifies the impact of model pretraining on the task.

Across architectures, pretrained embeddings substantially outperformed randomly initialized models under both linear probing and shallow nonlinear classification. At 8k context, GENERanno achieved an MCC of 0.979 compared to 0.418 for random embeddings, while megaDNA improved from 0.260 to 0.910 and Nucleotide Transformer v2 from 0.583 to 0.951 (Table 2 and Figure 3). In contrast, DNABERT-2 and Caduceus exhibited smaller gains from pretraining and correspondingly lower peak performance, indicating weaker representational power. Notably, the nonlinear classifiers provided only modest gains over linear probes, indicating that with strong sequence representations, this task is largely linearly separable.

Together, these results demonstrate that embedding strength is predictive, as models that show a small ΔMCC on this embedding test have weaker sequence representations and reduced downstream performance.

### Evaluating Peak Model Performance.

Each model was fine-tuned to evaluate its peak performance on the same test dataset used for the probing tasks. If a model was too large to fine-tune (Evo and Evo2) or was an autoregressive model (megaDNA), the best-performing model from the probing tasks was used. We replicated the experiment ten times and reported the average and standard deviation for each model (Table 3). Three different input sequence lengths (2k, 4k, 8k) were tested, and results were reported for each model up to its maximum context window length.

There are clear performance differences across architectures. At the longest input sequence length, EVO2 (0.966), megaDNA (0.936), GENERanno (0.936), and Nucleotide Transformer (0.930) exhibit near-perfect performance on this task. In contrast, Caduceus (0.820) and DNABERT-2 (0.677) had significantly lower performance. Models such as EVO2 and megaDNA, designed to use longer sequence lengths, appeared to extract more information from them, while the longer sequences provided limited benefit to other models. Consistent with earlier observations, the models with strong embedding structures in the probing experiments were the same models that achieved the highest fine-tuned performance.

### Evaluating Sources of Prediction Error.

#### Testing for GC-composition bias.

In order to test model dependency on GC-composition, we constructed a dataset where each sequence was shuffled to create a new sequence that had the same GC-composition. The models were then tested on this dataset to assess whether they exhibited GC-bias. Since the dataset had a 1:1 ratio of bacterial to phage sequences, and each sequence contained no meaningful genomic information, the ideal MCC score on the test set would be 0.00. This would indicate that the model would randomly predict the sequences to be either bacteria or phage. Under this control, the MCC values for all models ranged from 0 to 0.13, indicating that GC-composition accounts for only a small fraction of model performance, with DNABERT-2 and Nucleotide Transformer (v2) showing the highest MCC values, suggesting a slight GC-bias in these models.

#### Testing for Class Prediction Bias.

To evaluate model misclassification behavior, we constructed two controlled datasets: one containing only bacterial sequences to measure false-positive rates (phage predictions on non-phage sequences), and one containing only phage sequences to measure false-negative rates (missed phage predictions). False-positive rates (FPRs) using this controlled dataset varied substantially across models, ranging from 57.06% (Caduceus) to 2.26% (GENERanno). The false negative rates were in a tighter range with a high of 11.63% (Caduceus) and a low of 2.39% (GENERanno).

A comparison of the FPR with the FNR shows different patterns across the architectures. GENERanno and EVO2 have low and symmetric error rates, while Caduceus shows a strong bias in predicting phage, especially at lower sequence lengths. Several models show asymmetry in their FPR and FNR, which may be influenced by their pretraining datasets.

#### Testing Performance Across PHROG Functional Categories.

**Fig. 2.**
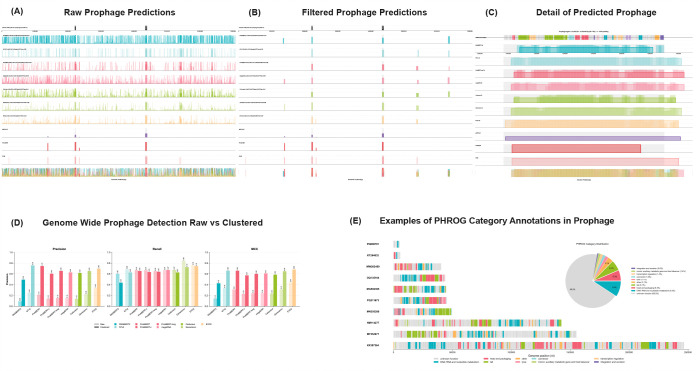
Genome-wide prophage detection and functional validation in LAMBDA. (A) Raw genome-wide prophage predictions across a representative bacterial chromosome, showing per-window model scores prior to post processing. (B) Prophage predictions after normalization, bidirectional smoothing, merging adjacent high-scoring regions, and filtering for score and length. The published ground-truth prophage locations are shown in black in the top row. Performance of PHASTER, Genomad and PIDE can be seen in the rows 9–11. The bottom row shows the combined signal of all gLMs. (C) Detailed view of a reference prophage region that includes predictions from all genomic language models shown against the PHROG functional category annotations. (D) Performance comparison of genome-wide detection before (*raw*) and after clustering (*filtered*), demonstrating improved precision and MCC with minimal loss of recall. (E) Examples of PHROG functional annotations on ten sample prophages from the test set.

**Fig. 3.**
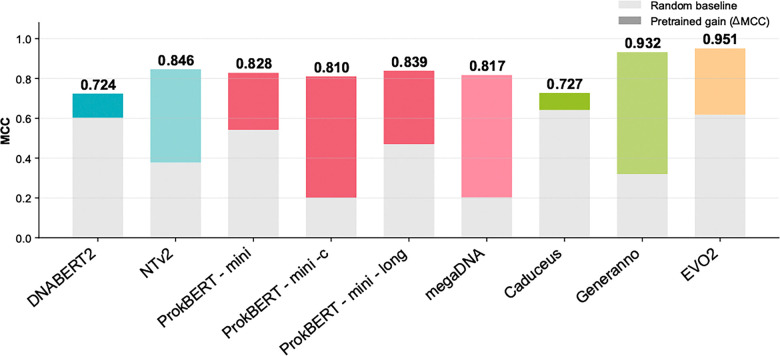
Benchmarking results of genomic language models on tests of foundational embedding strength. Embedding strength is quantified as ΔMCC, defined as the difference in Matthews Correlation Coefficient (MCC) between a probe trained on pretrained model embeddings and the same probe trained on embeddings from an identical architecture with randomly initialized weights. ΔMCC is shown in the figure in color in the stacked bar. The grey region is the performance of the model using random initialization. Higher ΔMCC values indicate greater representational gain attributable to pretraining.

To quantify the amount of phage signal within specific phage functional annotation groups, we evaluated each model on the same phage-only sequences, but segmented them by coding sequence (CDS) rather than by fixed length. Each CDS was annotated by Pharokka using the Prokaryotic Virus Remote Homologous Group Database (PHROG). PHROG groups are organized into nine high-level functional categories (Table 4). The category “unknown functions” contains sequences that are annotated with the term “hypothetical protein”, while the “other functions” category contains proteins that are known and annotated but do not fit into the other broad categories. Visualizations of the PHROG annotations for ten prophages from the test set are shown in Figure 2E. The performance of the models on this test evaluates how well each model’s sequence representation captures distinct classes of phage-associated proteins. Performance varied considerably across categories and models, with the “Head & Packaging” and “Tail” genes achieving the highest sensitivity (0.82). MegaDNA consistently underperformed, which we hypothesize is because the length of the CDS sequences is too short for its extremely long context window (96k). Caduceus appears to do well on this test, but this is an artifact of its very high false-positive rate.

### Genome-Wide Prophage Detection.

#### Extracting Prophage Signal from Raw Model Predictions.

To evaluate each model’s ability to scan a full bacterial genome to detect prophage locations, we assembled a reference dataset comprising 80 bacterial genomes with 386 verified integrated prophage locations. Each genome was scanned using fixed length sequences that overlapped by 50%, using each model’s best-performing model on that sequence length for inference. We report the false positive rates (FPR), the recall, and MCC scores on these predictions, which we label as “Raw” scores.

To extract coordinates for prophage detection, we use a prophage signal extraction algorithm that combines z-score normalization with bidirectional smoothing via an exponential moving average (EMA). We then merge closely spaced clusters and filter out prophage candidates by score and length. Similar clustering, merging and filtering algorithms are used in traditional prophage detection tools (26, 35, 39). These scores are the “Filtered” scores that can be viewed in Table 3, and a sample visualization of the raw scores compared to the filtered scores can be seen in Figure 2(A and B). A sample prophage detail showing the overlapping raw probability windows is shown in Figure 2C.

The genome-wide prophage detection task is considerably more challenging for genomic language models than the controlled segment classification. The false positive rate increased significantly across all models relative to the FPR in the controlled experiments. Per-genome normalization, and filtering significantly reduced false positives, increasing MCC scores across all models while only slightly decreasing recall (Figure 2D). After filtering, EVO2 achieved the highest overall performance (MCC 0.680) followed closely by ProkBERT-mini, Nucleotide Transformer, GENERanno and megaDNA. Consistent with earlier tests, DNABERT-2 and Caduceus had the weakest performance. In contrast to the segment-level tests, longer context lengths did not improve performance in the genome-wide prophage detection test, with most models having their best performance with the 2k sequence length.

#### Scanning for Novel, Unannotated Prophage Regions.

While defining prophage boundaries is inherently difficult, our scanning presented an opportunity to potentially detect novel, unannotated phage regions. Across the 80 bacterial genomes in our benchmark, we identified 305 candidate regions that were supported by between two and seventeen models (median of three models), and that did not overlap with any annotation in the published ground-truth dataset. To characterize these candidates, we performed a manual review of all 305 regions, evaluating each candidate for structural-gene, integration/excision, lysis, and annotation evidence (Supplementary Table 2). This review yielded 22 candidates classified as *likely phage*, 92 as *possible phage*, 113 as *weak phage signal*, 31 as *genomic island / ICE*, 16 as *genomic island*, and 31 as *unlikely phage*. Even within the 22 high-confidence candidates supported by at least 10 models, only nine were classified as likely phages, whereas 10 showed weak phage signal, six were classified as possible phages, one as unlikely phages, and one as a genomic island / ICE. A representative strong example within this set was a ~44 kb region in *Gordonia iterans* Co17 (candidate_39), supported by 14 models and classified as likely phages with eight head/packaging, 10 tail, three connector, and three lysis genes.

Across all 305 candidates, the predominant pattern was therefore ambiguous or non-phage mobile elements rather than intact prophages: 191 regions were classified as weak phage signal, genomic island/ICE, genomic island, or unlikely phage. These results indicate that most candidate regions flagged outside the ground truth are not spurious predictions but rather reflect genuine mobile genetic elements whose compositional and structural signatures overlap with those of prophages. This overlap between prophages and other mobile elements represents a fundamental challenge for all prophage detection methods and is a likely source of false-positive predictions across both gLMs and traditional tools. At the same time, the presence of 22 likely phage candidates absent from the published annotations suggests that the ground-truth dataset remains incomplete, and that the MCC scores we report are correspondingly conservative.

#### Probing EVO2 Latent Space with Sparse Autoencoders.

Sparse autoencoders (SAEs) are mechanistic interpretability tools that decompose the internal representations of a language model into a sparse set of interpretable features (40, 41). The recently published EVO2 model reports prophage detection as one of several biological features that EVO2 has learned using unsupervised learning. In their study, a sparse autoencoder (SAE) is used to extract information from the model’s internal neurons. One of the identified features, f/19746, was associated with prophage sequences across the prokaryotic domain. We used the EVO2 model, along with the trained SAE, to extract this signal for all genomes in our test set to evaluate its generalizability as a prophage detection tool.

We found that f/19746 activation varies considerably across genomes, firing cleanly in prophage regions of some genomes, but producing noisy baseline signal or sparse, non-prophage-associated activations in others. After applying our clustering algorithm, EVO2+SAE achieved competitive but lower performance than the EVO2 neural network classifier (average MCC of 0.636 vs. 0.680) (Figure S15).

### Comparison with Traditional and Protein-based Models.

A comparison with traditional prophage detection tools shows that genomic language model performance on this task lags slightly behind traditional methods and PIDE, a model built using protein language model embeddings (Table 5). EVO2 had the highest performance of the gLMs, with an MCC of 0.680, but this score was 0.11 lower than the model with the highest performance (Genomad). This performance was followed closely by ProkBERT-mini (0.658) and GENERanno (0.648). GENERanno had very high recall (0.723), but lower precision. The specificity (true negative rate) is very high across all models, indicating that the signal extraction algorithm consistently suppresses false-positive noise.

## Discussion

Standardized benchmarks that replicate unique domain challenges provide a foundation for the development and evaluation of new models. The LAMBDA benchmark provides this for DNA language models, presenting novel tasks that represent the unique challenges of detecting prokaryotic and viral sequence-level features in DNA. Our results demonstrate that previous studies showing randomly initialized models having similar or better performance than pretrained genomic language models (42) likely reflect insufficient benchmark difficulty rather than a lack of representational benefit from pretraining. The LAMBDA probing tasks quantify the contribution of pretraining and show that across nearly all architectures, pretrained embeddings significantly outperform their randomly initialized counterparts. This demonstrates that benchmark rigor is essential for evaluating the true sequence representational quality of genomic language models.

Our evaluation of gLM performance demonstrated that the quality of the training data and relevance to the domain were more important to performance than the scale of the models. Although EVO2, with seven billion parameters, achieved the highest performance among models, the second-highest performance was ProkBERT-mini, which has only 110 million parameters, but was trained on a large, carefully curated prokaryotic dataset. Similarly, GENERanno, which was also trained on prokaryotic sequence data, had a slightly lower MCC performance but higher recall than the other models. The weakest model performance (DNABERT-2, Caduceus) was observed in models trained entirely or predominantly on human DNA sequences. These results demonstrate that the domain and task orientation are stronger predictors of model performance. This has significant implications for the development of future gLMs, where the relative contributions of model size and training data curation need to be weighed against the computational and human costs of developing new models. As our results demonstrate, some tasks may require smaller, domain-specific models to achieve consistent and high performance.

We observed that false positive rates increased between two and ten times between our controlled tests and the genome-wide comparison. This demonstrates the challenges of biological sequence analysis, in which “hard negatives” take varied forms and contexts. These hard negatives are regions such as mobile elements, genomic islands, and degraded prophage remnants that share features with true prophage but are not complete prophage regions. While the training dataset was carefully curated, presenting controlled and clearly defined features, these regions lie closer to the decision boundary and are more likely to be misclassified. Our analysis of the novel candidate phage regions predicted by the gLMs revealed that there are still a large number of unidentified, likely phage regions within bacterial genomes. This highlights the challenges of phage identification and the importance of obtaining and curating high-quality benchmarking data.

Defining a complete ground truth for prophage content in bacterial genomes is inherently difficult. The annotation of prophages depends on the tools and reference databases available at the time of curation. Further complicating this task is the fact that the boundaries between prophages, integrative conjugative elements (ICEs), pathogenicity islands, and other mobile genetic elements are not always clear-cut, as these elements often share core integration and mobilization machinery. This overlap between prophages and other mobile elements poses a fundamental challenge for all prophage detection methods and is likely a source of false-positive predictions in both gLMs and traditional tools. At the same time, the detection of nearly complete prophages in the *Y. pestis* genomes demonstrates that the ground-truth dataset itself is incomplete, needing additional experimental work to identify and characterize phages. This also implies that the MCC scores that we report are likely conservative, being impacted by the incomplete knowledge of phages in bacterial genomes.

Our analysis also provides an opportunity to evaluate the potential impact of new approaches within the field. For example, our analysis of the EVO2 sparse autoencoder output indicated that SAEs represent a promising avenue for future research, but that the EVO2+SAE feature f/19746 does not generalize uniformly across the prokaryotic domain. We propose two possible explanations for this limited generalizability, insufficient domain-specific training data and distributed encoding of the prophage signal. The extracted feature can only reflect what the model has learned during training, and since a large portion of the EVO2 dataset was focused on regulatory information in the eukaryotic domain, the model may be undertrained on specific bacterial taxa. Second, the prophage signal may not be fully encoded in a single feature from one layer but could be distributed across a circuit of interacting neurons, only one of which has been identified. Circuit-level representations have been demonstrated in transformer language models (40, 43–45), suggesting that a more comprehensive circuit-level analysis may be needed to capture the full prophage signal across the entire prokaryotic domain.

## Conclusion

We conclude that current genomic language models capture meaningful DNA sequence representations relevant to the prophage detection annotation task, although homology-based tools still achieve higher performance on this task. LAMBDA addresses a critical gap in existing benchmarks by providing specific metrics that reveal where models fail and moves beyond classification tasks toward genome-wide screening for specific sequence patterns and motifs. This rigorous framework highlights the current limitations of genomic language models relative to their protein counterparts. The consistent relationship we observed between the representational power of embeddings and downstream accuracy suggests that improvements in pretraining and training data curation will translate into meaningful gains on genomic annotation capabilities.

### Limitations.

Although we used the batch sizes and learning rates recommended by the authors for each model, the potential benefits of extensive hyperparameter tuning for model performance remain to be explored. Similarly, signal extraction methods can significantly affect final performance metrics, and more research is needed to identify the most effective and consistent approaches. Additionally, as our manual review of 305 candidate regions demonstrates, the boundaries between prophages, ICEs, and other mobile genetic elements are often ambiguous, and the ground-truth dataset likely remains incomplete despite expert curation. This overlap between prophage and non-phage mobile element signatures represents a fundamental challenge for all detection methods and a source of both false-positive and false-negative predictions. Additional analyses, including systematic comparisons with mobile genetic element datasets, are needed to better characterize gLM limitations and inform future improvement strategies.

## Materials and Methods

### LAMBDA Benchmark Dataset Construction.

The LAMBDA benchmark dataset was constructed to train and evaluate genomic language models for prophage detection by combining curated phage genomes (positive class) with high-quality bacterial genomes (negative class), ensuring balanced representation and preventing data leakage between train, development, and test splits.

#### Data Sources.

Complete phage genomes were obtained from the INPHARED (INfrastructure for a PHAge REference Database) database (14 Apr 2025 release) (46). Clusters with 95% average nucleotide identity (ANI) were generated using vclust (47), and one representative genome per cluster was selected, yielding 8,684 genomes for downstream processing. Gene annotation was obtained using Pharokka (48). Bacterial representative genomes were downloaded from the Genome Taxonomy Database (GTDB) (49). The genomes were filtered for high quality based on completeness (≥95%) and contamination (≤5%) using CheckM (50), producing a set of 67,193 assemblies. One genome per GTDB genus was randomly subsampled to ensure broad taxonomic coverage, resulting in 15,865 genomes.

#### Bacterial contamination filtering via BLAST.

To remove bacterial genomes that contained phage sequences, we performed sequence similarity searches between the curated phage set and the bacterial representatives using BLAST+ (Basic Local Alignment Search Tool) with parameters set to a minimum alignment length of ≥200 base pairs (bp), minimum identity ≥ 90%, and max_target_seqs=2000. Bacterial genomes with significant hits to phage sequences were identified as contaminated and excluded from the final negative class. This BLAST filtering step created a bacterial dataset that had no detectable phage sequences above the specified thresholds.

#### Segment Subsampling.

Following filtering, genomes were subsampled into fixed-length segments of 2000 nt, 4000 nt, and 8000 nt to create three datasets. For phages, segments were sampled at random, non-overlapping positions at a rate of one segment per 10 kbp, with a minimum of one sample per genome. The bacterial segments were sampled uniformly across genomes to match the total number of phage segments to achieve a 1:1 ratio of phage to bacterial segments.

#### Data Splitting and Data Leakage Prevention.

To avoid data leakage between the dataset partitions, phage genomes were split based on the vclust clusters, so that each cluster was in only one partition. Bacterial genomes were split by GTDB genus with only one cluster per partition. The data split between train, dev and test dataset was 8:1:1.

#### GC-Control Dataset.

Every sample in the test dataset had its nucleotide order shuffled to create a control dataset with sequences that preserved sequence length and GC content while destroying higher order sequence patterns.

#### Class Prediction Control Datasets.

To estimate false positive rates on bacterial seqeunces, we created a bacteria-only benchmark consisting of 100 taxonomically balanced bacterial genomes drawn from the prophage-filtered GTDB test set. Genomes were selected to maximize breadth. To estimate the false negative rates on phage sequences, we constructed a phage-only dataset from the test split (869 genomes).

#### PHROG Category Prediction Control Dataset.

We created a second phage-only dataset using the 869 phage genomes in the test set, annotated with PHROG categories using Pharokka. In this PHROG dataset, each sample was a coding sequence (CDS).

#### Genome-Wide Evaluation Set.

To test the models against the challenge of scanning a full bacterial genome and detecting the presence of prophages, we assembled a reference dataset with published datasets that contain expert annotated prophage locations within bacterial genomes. We include the gold standard dataset published by Casjens et al. (24), which has been widely used by many prophage prediction programs, including PHASTER (26), as well as 23 additional prophage locations that were manually annotated by the authors of PHASTER. Another 63 reference locations were taken from Gauthier et al. (51), to add diversity. The final dataset spans 80 bacterial genomes representing 47 species across four phyla, which contain 386 annotated prophage locations. The viral taxonomy was obtained via Pharokka and identified 33 phage genera in two viral classes (Caudoviricetes and Faserviricetes), though the majority remain unclassified at the genus level (Figure S11). The reference bacterial accession numbers and the start and end positions for each predicted phage are provided (Table S1).

#### Prophage Signal Extraction Algorithm.

The initial step used majority voting to collapse the overlapping windows into a single score for each window. After observing that the baseline level of prophage signal depends on the genome, we designed an algorithm that normalizes per-segment scores on a per-genome basis. We evaluated four normalization strategies, no normalization (raw probabilities), z-score normalization ((score-mean)/std), robust z-score normalization ((score-median)/MAD), and median subtraction (score-median), with z-score normalization having the most consistent results. Following normalization, we applied a bidirectional exponentially-weighted mean (EWM) smoothing. Normalized scores were then thresholded to produce a binary phage or non-phage call for each segment. Small gaps were merged into candidate prophage regions, and a length filter was applied to retain only candidates that exceeded the threshold length. To find the optimal hyperparameters for this algorithm for each model, we conducted a hyperparameter search of all parameters in every model (Figure S14, Table S4) The optimal hyperparameters for each model were used for the final MCC scores.

#### Candidate Prophage Screening.

To identify potentially unannotated prophage regions, we scanned all 80 genomes for model predictions that do not overlap any ground truth region. Non-ground-truth predictions from all seventeen models were pooled, and overlapping predictions across models were merged into consensus candidate regions. Candidates supported by two or more models were retained. Each candidate was flagged as “novel” if it was missed by both PHASTER and Genomad. Each candidate was manually annotated by extracting gene content from NCBI reference annotations and classifying each region as containing or lacking phage hallmark genes. “Confident phages” were defined as those with ≥10% structural genes with head/tail, “possible phages” with 5−10% structural, some evidence, “weak signal” was defined as having only 1–2 structural genes, likely degraded prophage. Those defined as “not phages” contained sequences that were either genomic islands, integrative conjugative elements (ICEs), or unlikely phages (Supplementary Table 2).

#### Comparison with Traditional and Protein-based Models.

We compared the predictions from the genomic language models to six traditional prophage detection tools and one protein language model-based tool. PIDE (52) is a recently published prophage detection tool that, to our knowledge, is the first published model built on protein language model embeddings. PHASTER is a widely used web server that identifies prophages through similarity to curated phage databases and structural feature annotation. We used the PHASTER Docker image for our screening and to calculate the per-genome processing time. Genomad (35) integrates homology searches with a deep neural network classifier to detect viral sequences in microbial genomes. We also included VI-BRANT (31), Phigaro (39), Phispy (28), and VirSorter2 (33) in our comparisons. These tools collectively span homology-based, feature-based, and deep learning paradigms, providing a broad baseline for comparison.

## Supplementary Material

Supplement 1

Supplement 2

## Figures and Tables

**Fig. 1.**
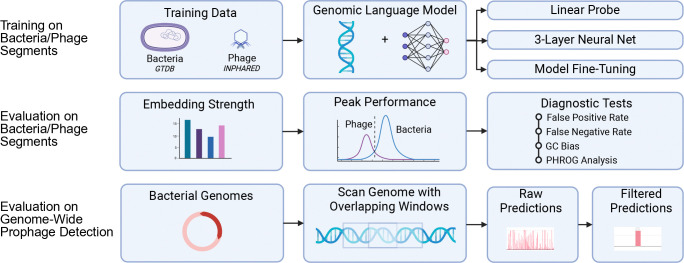
Overview of the LAMBDA benchmark for evaluating genomic language models. Models are trained on bacteria and phage genome segments (GTDB and INPHARED) and evaluated across three complementary axes: (i) embedding strength using linear probes and a shallow neural network over frozen embeddings, (ii) peak performance via fine-tuning, and (iii) diagnostic tests to determine sources of error (false positive/negative rates, GC bias, and PHROG-based functional analysis). Genome-wide evaluation is performed by using the pre-trained models to scan complete bacterial assemblies with overlapping windows, producing raw predictions that are subsequently filtered to identify high-confidence prophage regions.

**Table 1.** Genomic language models evaluated in the LAMBDA benchmark. For each model, we report the architecture, number of parameters, tokenization strategy, and training corpus. Models span a range of scales and pretraining regimes, including bacteria-specific, bacteriophage-specific and multi-organism training datasets.

Model	Architecture	Parameters	Max Context Length	Tokenization Method	Dataset

Caduceus (36)	bi-Mamba	1M	8K	char	Human DNA (Hg38)
ProkBERT (37)	BERT	110M	4K	k-mer	Bacteria + Virus
DNABERT-2 (6)	BERT	117M	2.5k	bpe	Multi-species
megaDNA (14)	GPT	145M	96K	char	Bacteriophage
GENERanno (13)	BERT	500M	8K	char	Bacteria
Nucleotide Transformer v2(7)	BERT	500M	6k	k-mers	Multi-species
EVO2 (12)	StripedHyena	7B	1M	char	OpenGenome2 (multi-species)

**Table 2.** Linear Probe and 3-Layer Neural Network Classification Results (MCC)

Model	Length	Linear Probe		3-Layer NN	
	
		Model	Random	Model	Random

DNABERT2	2k	0.724	0.603	0.777	0.678
DNABERT2	4k	0.734	0.650	0.794	0.708

Nucleotide Transformer v2	2k	0.846	0.378	0.876	0.368
Nucleotide Transformer v2	4k	0.924	0.491	0.936	0.522
Nucleotide Transformer v2	8k	0.951	0.583	0.958	0.650

Caduceus	2k	0.727	0.642	0.742	0.645
Caduceus	4k	0.788	0.685	0.789	0.651
Caduceus	8k	0.835	0.723	0.832	0.716

ProkBERT-mini	2k	0.828	0.542	0.705	0.380
ProkBERT-mini-c	2k	0.810	0.202	0.679	0.198
ProkBERT-mini-long	2k	0.839	0.470	0.875	0.336

megaDNA	2k	0.817	0.203	0.867	0.308
megaDNA	4k	0.874	0.207	0.914	0.368
megaDNA	8k	0.910	0.260	0.936	0.404

GENERanno	2k	0.932	0.320	0.936	0.302
GENERanno	4k	0.967	0.380	0.966	0.363
GENERanno	8k	0.979	0.418	0.979	0.396

EVO2	2k	0.951	0.618	0.944	0.258
EVO2	4k	0.964	0.662	0.964	0.344
EVO2	8k	0.974	0.704	0.966	0.622

**Table 3.** Detailed performance of genomic language models across fragment-level diagnostic tests and genome-wide prophage detection in the LAMBDA benchmark. Fragment classification metrics assess model bias and error modes using GC-bias (Matthews correlation coefficient, MCC, on nucleotide-shuffled sequences that preserve GC composition; ideal ≈0), false positive rate (FPR; bacteria-only), and false negative rate (FNR; phage-only). Peak MCC denotes the maximum MCC achieved on the full fragment classification test set. Genome-wide evaluation is performed by scanning complete bacterial genomes with overlapping windows to generate per-segment predictions. “Raw” results correspond to unprocessed predictions at a fixed threshold (0.5). “Filtered” results apply postprocessing, including per-genome z-score normalization, bidirectional smoothing, density-based clustering, and size filtering, to identify contiguous prophage regions. Genome-wide FPR, recall, and MCC are computed at the region level and macro-averaged across genomes.

		Fragment Classification				Genome-wide Prophage Detection					
		Diagnostic Tests				Raw			Filtered		
			
Model	Segment Length	GC-bias *MCC*	FPR	FNR	Peak *MCC*	FPR (%)	Recall (%)	MCC	FPR (%)	Recall (%)	MCC
DNABERT-2	2k	0.101	5.18%	10.34%	0.652	26.59	59.92	0.138	1.78	46.73	0.391
	4k	0.112	6.80%	14.59%	0.677	16.56	45.92	0.147	1.46	39.33	0.371

Nucleotide Transformer v2	2k	0.074	4.12%	3.33%	0.893	15.47	68.29	0.332	0.75	65.25	0.647
	4k	0.109	25.47%	3.60%	0.894	22.16	75.79	0.274	1.65	61.53	0.541
	8k	0.110	14.85%	9.79%	0.930	**3.07**	49.90	0.441	1.01	54.83	0.558

Caduceus	2k	0.076	57.06%	8.86%	0.832	19.32	67.17	0.232	1.12	62.66	0.577
	4k	0.056	29.97%	6.78%	0.840	24.76	69.80	0.221	1.10	57.33	0.542
	8k	0.063	23.55%	11.63%	0.820	14.85	62.97	0.330	1.80	58.43	0.521

ProkBERT-mini	2k	(0.010)	2.68%	8.70%	0.935	12.23	66.76	0.310	**0.63**	65.10	0.658
ProkBERT-mini-c	2k	0.092	3.88%	6.96%	0.924	18.71	66.60	0.227	1.57	64.59	0.564
ProkBERT-mini-long	2k	0.047	3.42%	9.67%	0.897	17.49	64.88	0.239	1.12	63.62	0.599

megaDNA	2k	0.049	1.04%	9.50%	0.867	18.23	66.74	0.240	0.74	60.61	0.602
	4k	0.120	0.83%	9.49%	0.914	12.23	64.49	0.329	1.03	67.47	0.633
	8k	0.120	2.30%	10.95%	0.936	9.18	59.37	0.400	0.86	61.19	0.621

GENERanno	2k	0.004	2.26%	2.39%	0.926	28.58	**84.10**	0.314	1.21	72.26	0.648
	4k	0.133	2.37%	2.56%	0.956	34.65	81.77	0.284	2.01	71.26	0.609
	8k	0.001	4.65%	2.48%	0.936	31.95	78.89	0.340	7.23	69.10	0.511

EVO2	2k	0.012	4.40%	3.07%	0.944	12.46	75.76	0.439	1.18	**74.15**	**0.680**
	4k	0.090	2.67%	2.19%	0.964	14.92	76.10	0.450	1.23	66.86	0.628
	8k	0.123	3.45%	2.03%	0.966	15.80	73.97	**0.452**	1.49	64.61	0.591

**Table 4.** Phage gene detection sensitivity stratified by PHROG functional categories across genomic language models. PHROG (Phage Orthologous Groups) categories represent curated functional groupings of phage genes based on sequence homology. Evaluation is performed on a fully annotated phage dataset, where each gene is assigned to a PHROG category. “% of Total” indicates the fraction of annotated phage genes belonging to each category in this dataset. Sensitivity is defined as the fraction of annotated phage genes in each category that the model correctly identifies as phage. Results are reported for six models, with categories ordered by average sensitivity across models (highest to lowest).

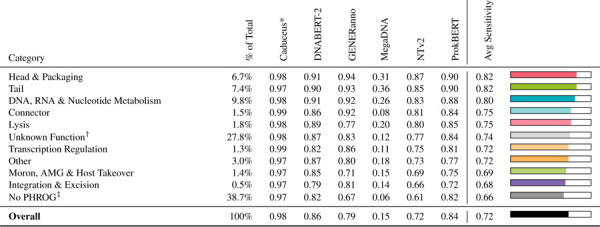

* Very high sensitivity can be driven by a high false positive rate

† Matched to a PHROG entry but no known biological function assigned

‡ No match to any entry in the PHROG database

**Table 5.** Comparison of genomic language models with traditional prophage detection tools. Models are evaluated on genome-wide prophage detection and ranked by Matthews Correlation Coefficient (MCC). Shaded rows indicate genomic language models (gLMs) fine-tuned for prophage detection (blue); a protein language model-based approach (pink) is included for reference. Traditional tools (unshaded) represent current state-of-the-art methods for prophage identification.

Model	Precision	Recall	Specificity	FPR	F1	MCC

geNomad	0.761	0.873	0.991	0.009	0.786	0.794
PHASTER	0.764	0.855	0.989	0.011	0.780	0.786
VIBRANT	0.728	0.759	0.993	0.007	0.722	0.726
Phigaro	0.839	0.662	0.996	0.004	0.709	0.721
PIDE^‡^	0.647	0.844	0.984	0.016	0.707	0.715
EVO2^†^	0.694	0.742	0.988	0.012	0.665	0.680
ProkBERT-mini^†^	0.743	0.651	0.994	0.006	0.640	0.658
NTv2^†^	0.756	0.627	0.995	0.005	0.644	0.658
GENERanno^†^	0.651	0.723	0.988	0.012	0.631	0.648
megaDNA^†^	0.627	0.671	0.988	0.012	0.595	0.609
ProkBERT-mini-long^†^	0.654	0.636	0.989	0.011	0.579	0.599
PhiSpy	0.604	0.668	0.982	0.018	0.567	0.585
Caduceus^†^	0.616	0.625	0.990	0.010	0.567	0.583
ProkBERT-mini-c^†^	0.603	0.632	0.987	0.013	0.551	0.572
PhageBoost	0.468	0.680	0.966	0.034	0.494	0.513
DNABERT-2^†^	0.492	0.436	0.991	0.009	0.413	0.425
VirSorter2	0.289	0.594	0.954	0.046	0.375	0.387

† Genomic Language Models

‡ Protein Language Model

## Data Availability

All datasets used in this study were derived from publicly available sources. Bacterial genomes were obtained from the Genome Taxonomy Database (GTDB), and phage genomes were obtained from the INfrastructure for a PHAge REference Database (INPHARED). The processed datasets used in the LAMBDA bench-mark, including training, evaluation, and diagnostic test sets, are available for download from https://doi.org/10.5281/zenodo.19236553. Instructions for dataset access and reconstruction are available at the LAMBDA repository: https://github.com/leannmlindsey/LAMBDA.

## Impact Statement

Our work provides a benchmark for detecting prophage regions within bacterial genomes, ideally driving the development of new genomic language models capable of handling diverse genomic datasets. The potential impacts of this work in biomedical research are wide-ranging, having implications for evolutionary analyses, bioengineering, and the development of novel therapeutics. While we anticipate that future research will have positive impacts on society and human health, any bioengineering, genomic, or health-focused research should be conducted ethically and consider potential negative outcomes.

## Code Availability

All code required to reproduce the LAMBDA benchmark, including model training, evaluation, and analysis pipelines, is available through a central repository. https://github.com/leannmlindsey/LAMBDA.

An interactive web-based visualization tool is available to visualize the genome-wide prophage predictions on the test set, including results from the EVO2 + sparse autoencoder (SAE) analysis. https://leannmlindsey.github.io/lambda-benchmark/.
