# Supplementary material for: LAMBDA: A Prophage Detection Benchmark for Genomic Language Models
Source: bioRxiv. 2026 Mar 26:2026.03.26.714501. Preprint. [Version 1] doi: 10.64898/2026.03.26.714501 (PMC13041943; doi:10.64898/2026.03.26.714501)
Supplement: Supplement 2 [file media-2.pdf]

Supplementary Figures Legend

Figure S1: Pretrained Embedding Performance Compared to Random for 2k, 4k, 8k Datasets

Figure S2: Relationship between CheckV-derived prophage quality metrics and per-genome model performance (MCC)

Figure S3: Case study of clean genome-wide prophage detection in *Mycobacterioides abscessus with MCC score 0.91*

Figure S4: Case study of genome-wide prophage detection in *Escherichia coli* K-12 with MCC score 0.15

Figure S5: Case study of genome-wide prophage detection in *Pseudomonas aeruginosa* NCGM2.S3 with MCC score 0.67

Figure S6: Detailed view of Prophage region 1 (1,697,680–1,740,560 bp) in *Pseudomonas aeruginosa* NCGM2.S3 with PHROG functional annotations

Figure S7: Model Performance Stratified by Bacterial Genome Taxonomy (Phylum)

Figure S8: Model Performance Stratified by Bacterial Genome Taxonomy (Class)

Figure S9: Model Performance Stratified by Bacterial Genome Taxonomy (Order)

Figure S10: Model Performance Stratified by Bacterial Genome Taxonomy (Genus)

Figure S11: Model Performance Stratified by Viral Lineage

Figure S12: Model Performance compared to MASH distance to Nearest INPHARED Phage

Figure S13: Per-model Detection Rate for Known vs. Novel Prophages

Figure S14: Hyperparameter Grid-Search for Prophage Signal Extraction Algorithm

Figure S15: Genome-wide comparison of EVO2 and EVO2+SAE activations

Figure S16: Website to Visualize the LAMBDA predictions

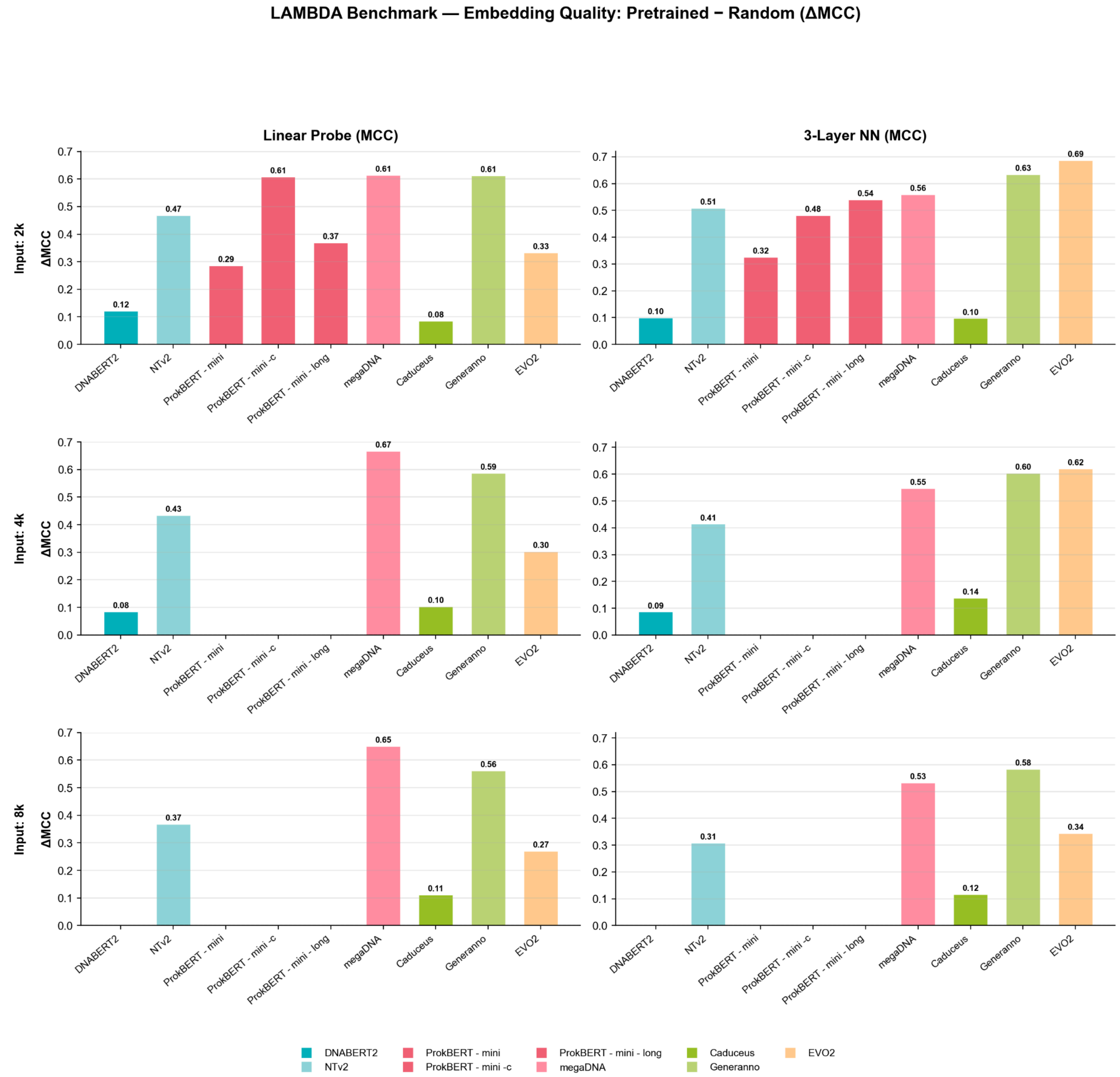

Figure S1: Pretrained Embedding Performance Compared to Random for 2k, 4k, 8k Datasets. Pretrained embedding strength is defined as the difference in MCC between pretrained embeddings and embeddings from the same model with randomly initialized weights (ΔMCC), where larger ΔMCC values indicate a stronger contribution from pretraining. Models were only evaluated on sequences that fit in their context window.

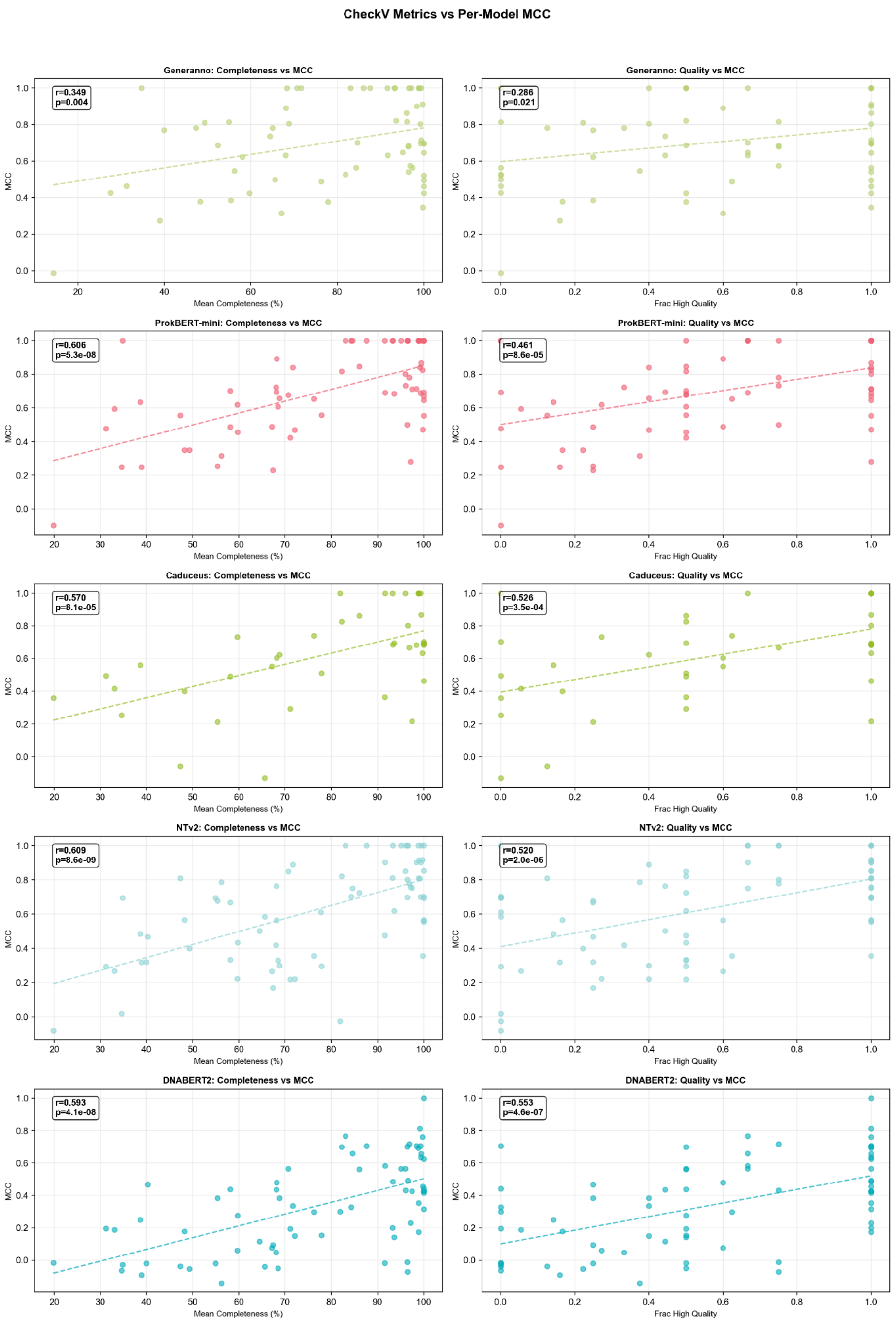

Figure S2: Relationship between CheckV-derived prophage quality metrics and per-genome model performance (MCC). For each model, mean CheckV completeness (left) and the fraction of high-quality prophages (right) are plotted against genome-level MCC. Dashed lines indicate linear regression fits, with Pearson correlation coefficients (r) and corresponding p-values annotated. All models exhibit positive correlations, indicating that higher predicted prophage quality is associated with improved genome-level classification performance.

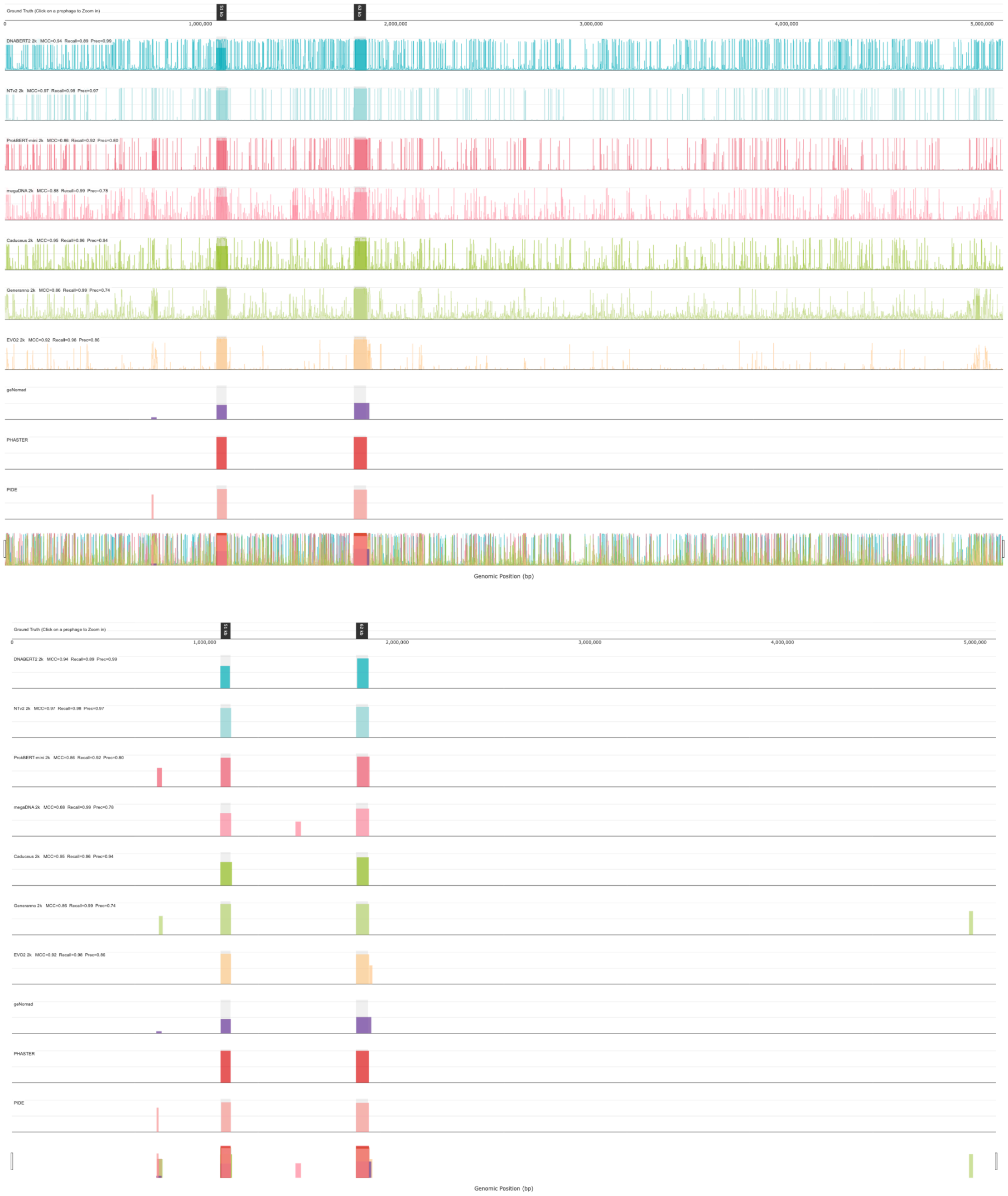

Figure S3: Case study of clean genome-wide prophage detection in *Mycobacterioides abscessus with MCC score 0.91.* Raw embedding-derived signal (top), and predicted regions (bottom). In this example, strong, localized peaks in the raw model signal align closely with annotated prophage loci, producing sharp, well-delimited predictions with minimal background activation across the rest of the genome. Similar interactive visualizations are available for all LAMBDA test genomes at https://leannmlindsey.github.io/lambda-benchmark/.

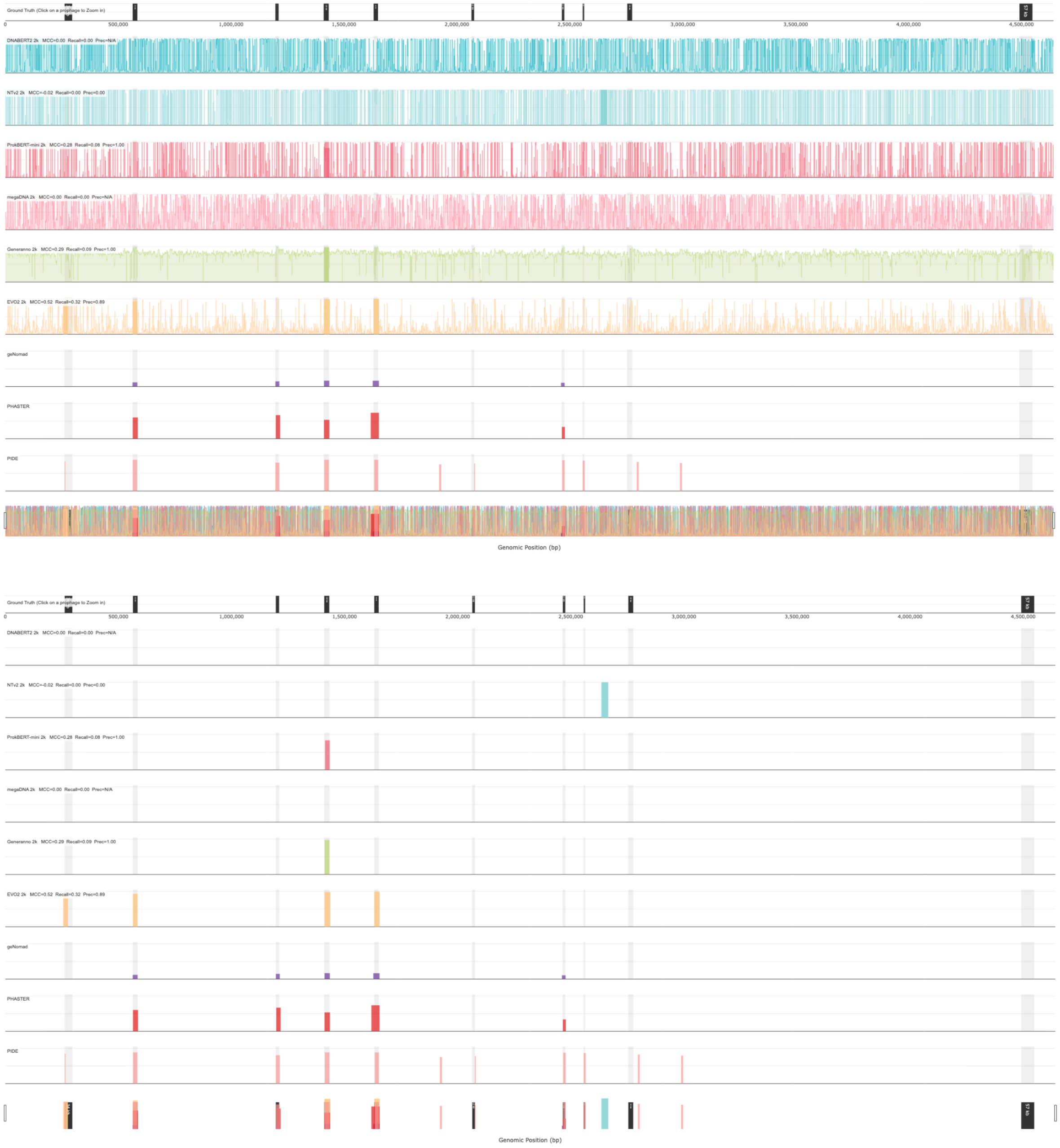

Figure S4: Case study of genome-wide prophage detection in *Escherichia coli* K-12 with MCC score 0.15. Raw embedding-derived signal (top), and predicted regions (bottom). In contrast to the *Mycobacterioides abscessus* example, signal across the genome is more diffuse, with fewer sharply localized peaks. Predicted regions partially overlap annotated loci but exhibit increased background activation, illustrating a more challenging genomic context and reduced signal-to-noise. Similar interactive visualizations are available for all LAMBDA test genomes at https://leannmlindsey.github.io/lambda-benchmark/.

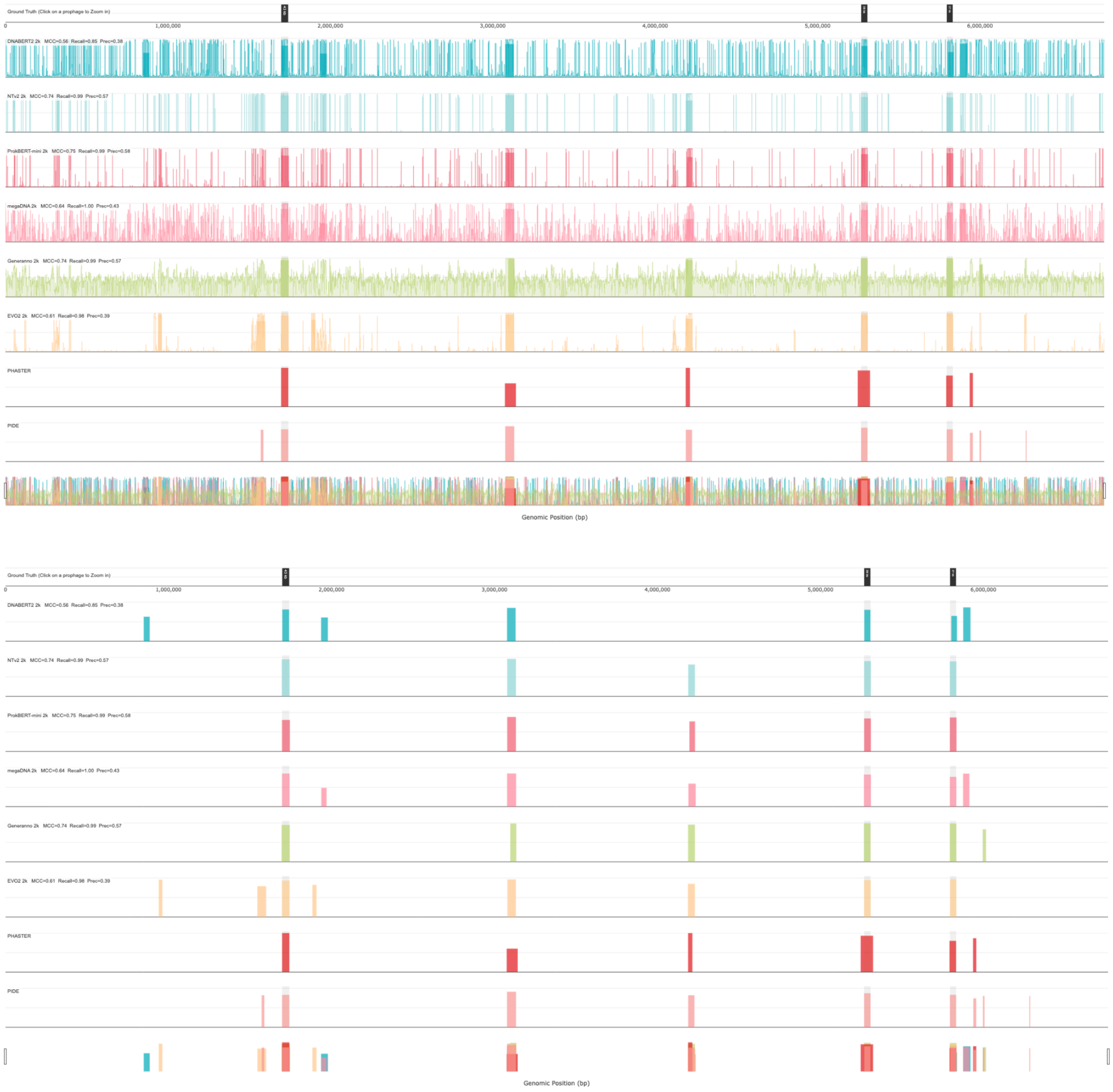

Figure S5: Case study of genome-wide prophage detection in *Pseudomonas aeruginosa* NCGM2.S3 with MCC score 0.67. Raw embedding-derived signal (top), and predicted regions (bottom). Two candidate prophage regions are consistently identified by both genomic language models and traditional prophage-detection tools (bottom tracks), with concordant peaks in the raw embedding signal. These shared loci highlight cross-method agreement despite more diffuse background activation elsewhere in the genome. Similar interactive visualizations are available for all LAMBDA test genomes at https://leannmlindsey.github.io/lambda-benchmark/.

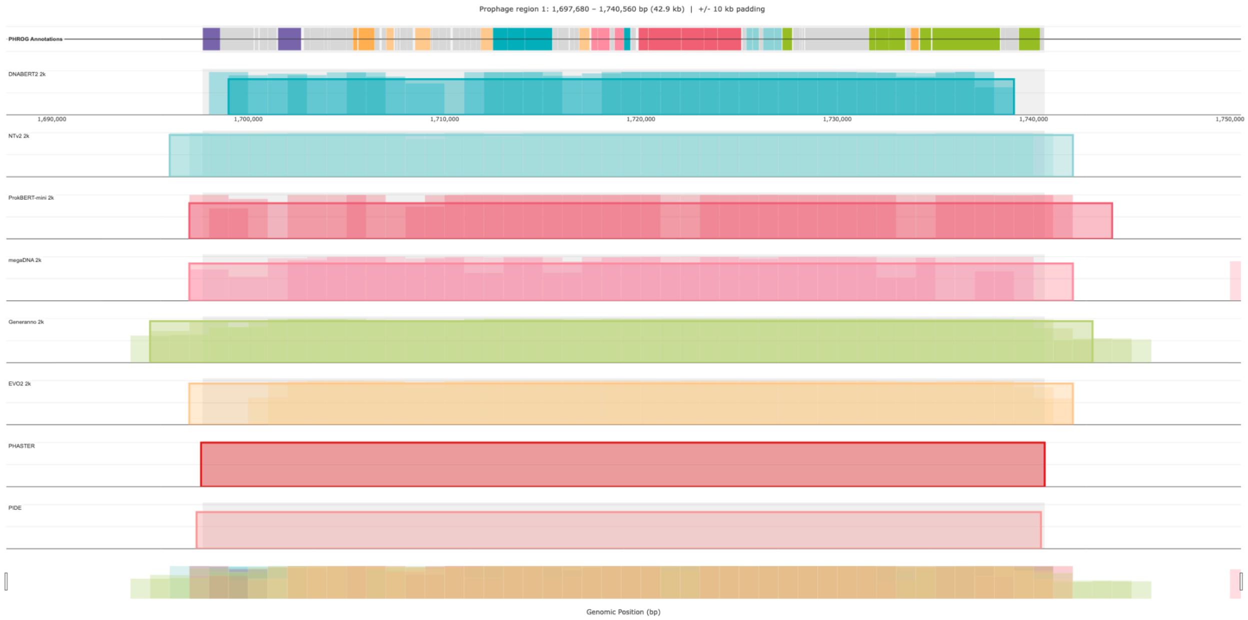

Figure S6: Detailed view of Prophage region 1 (1,697,680–1,740,560 bp) in *Pseudomonas aeruginosa* NCGM2.S3 with PHROG functional annotations. Zoomed-in visualization of prophage region 1 (±10 kb padding) showing PHROG functional categories across genes (top track) alongside genomic language model predictions and traditional tool calls. Embedding-derived signal forms a contiguous, high-confidence region that aligns closely with PHROG-annotated structural and replication-associated phage genes, with boundaries largely consistent with PHASTER and PIDE predictions. Similar interactive visualizations are available for all prophage regions in the benchmark at https://leannmlindsey.github.io/lambda-benchmark/

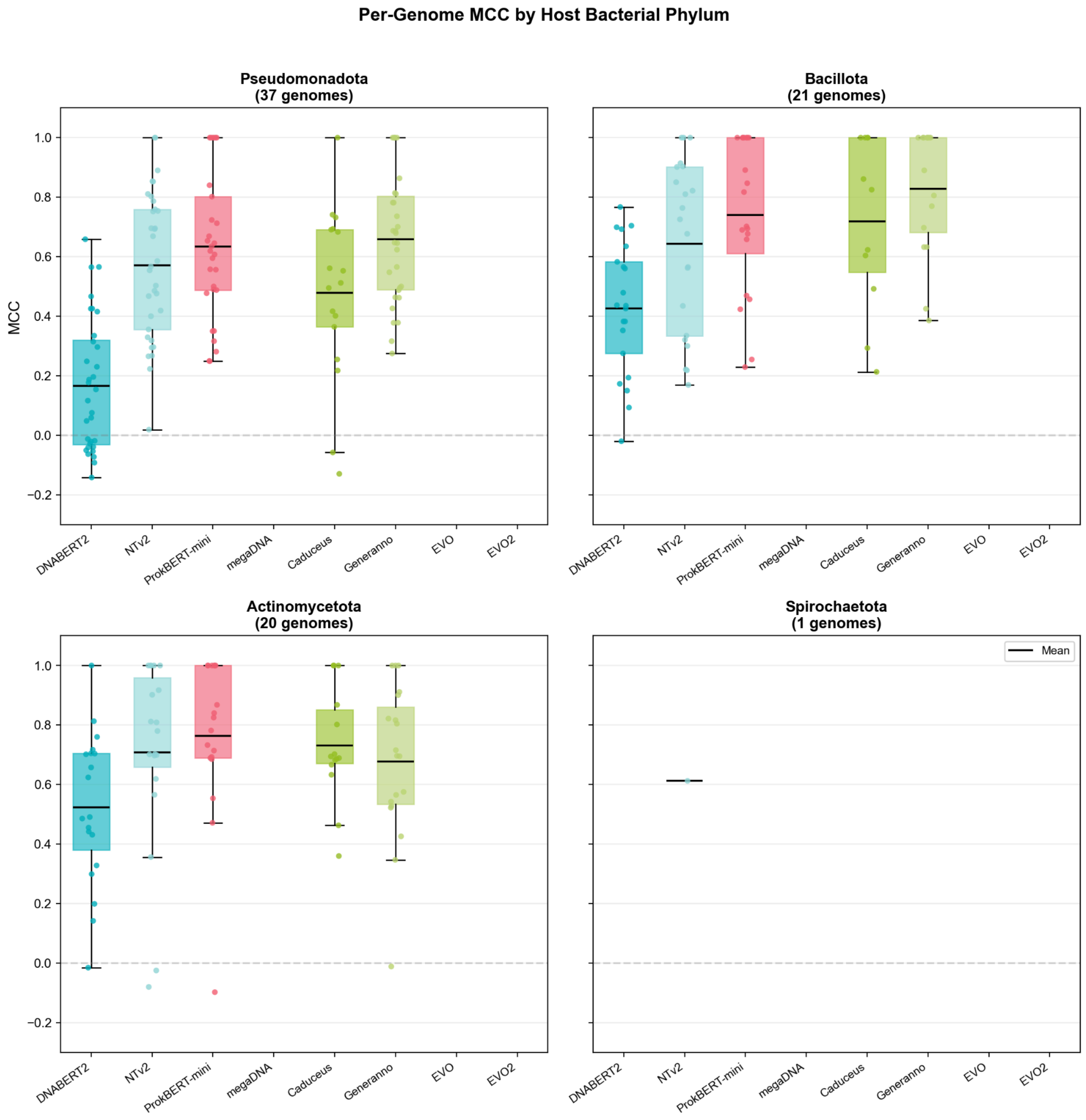

Figure S7: Model Performance Stratified by Bacterial Genome Taxonomy (Phylum). Per-genome MCC of prophage detection by host bacterial phylum. Each panel shows one phylum, with boxplots for each model (2k input). Points represent individual genomes. Black lines indicate means.

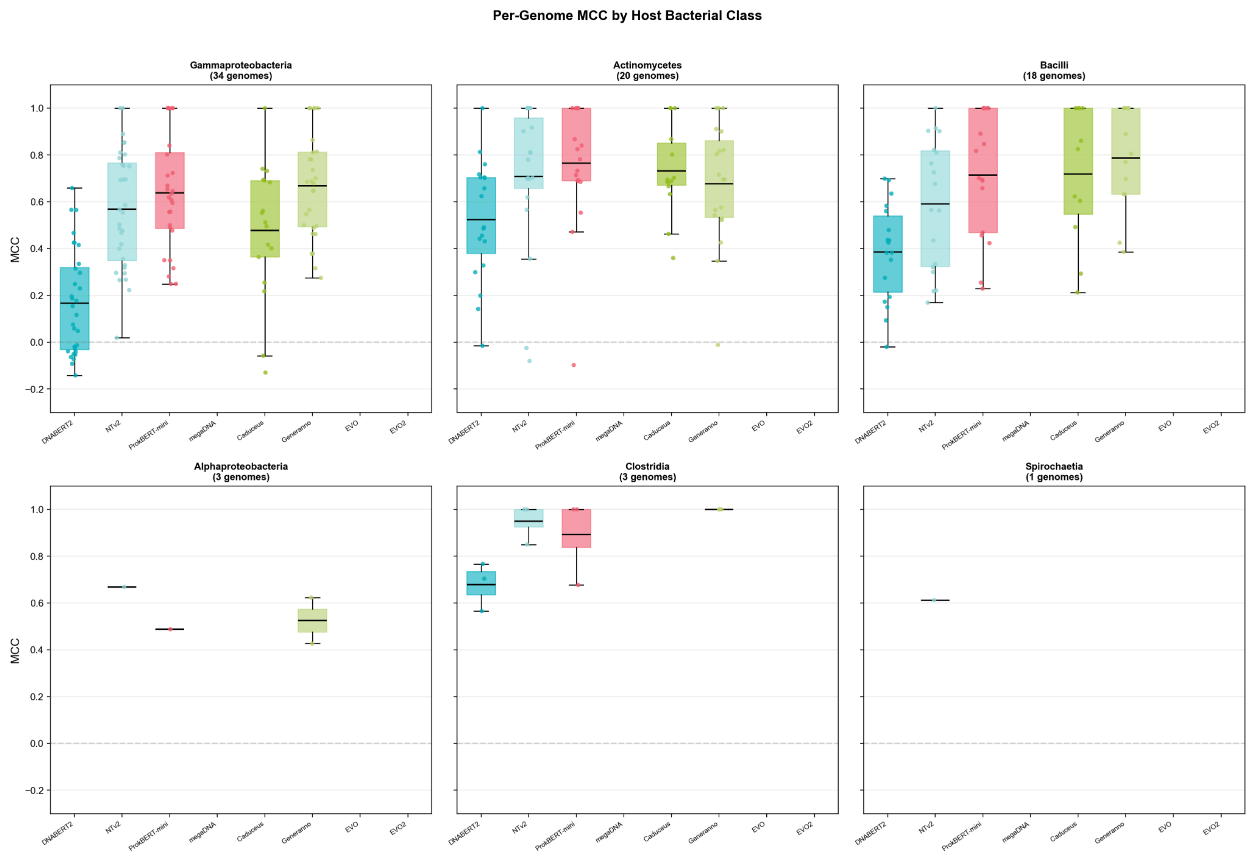

Figure S8: Model Performance Stratified by Bacterial Genome Taxonomy (Class). Per-genome MCC of prophage detection by host bacterial class. Each panel shows one phylum, with boxplots for each model (2k input). Points represent individual genomes. Black lines indicate means.

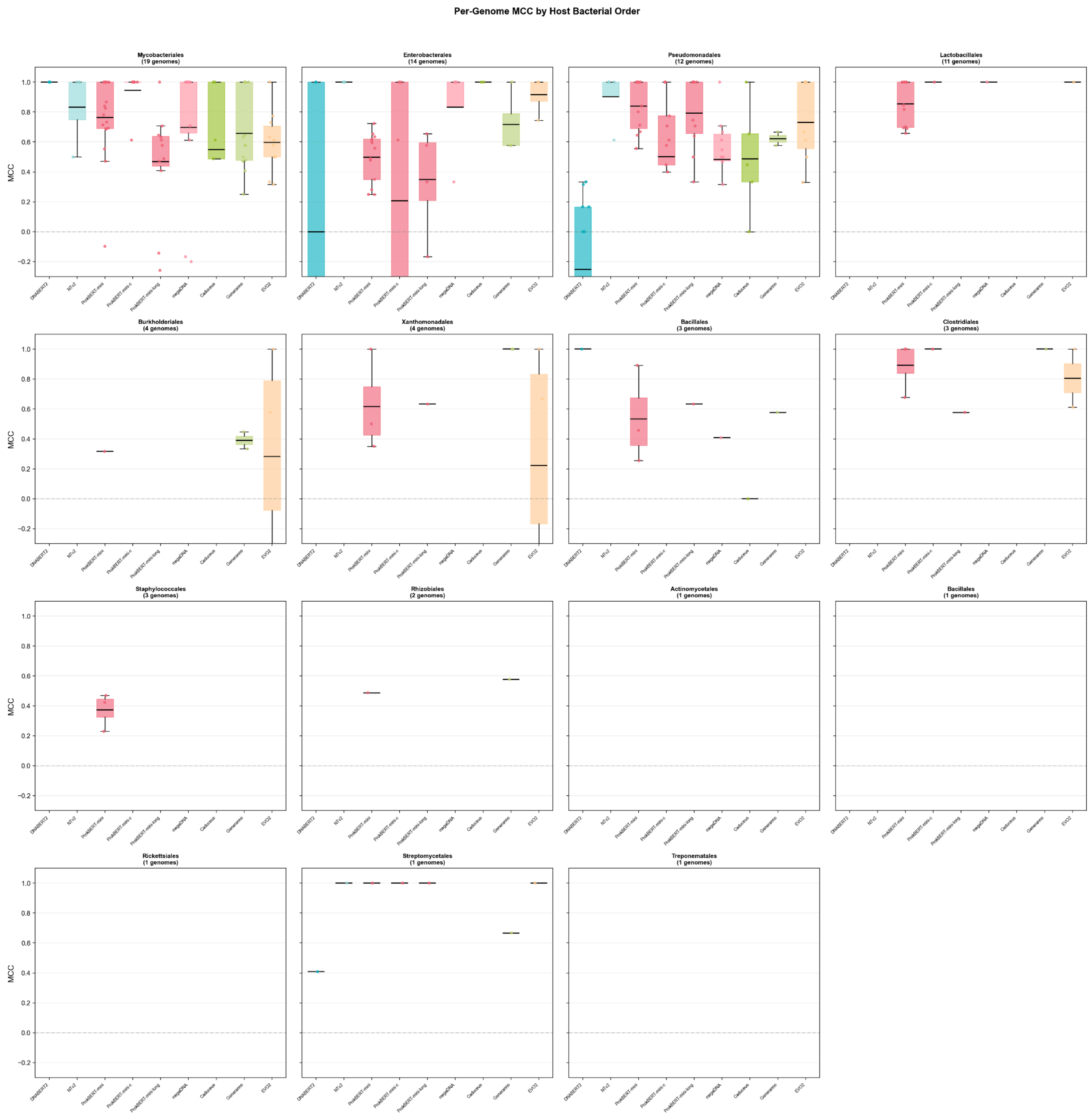

Figure S9: Model Performance Stratified by Bacterial Genome Taxonomy (Order). Per-genome MCC of prophage detection by host bacterial order. Groups are sorted by number of genomes (descending). Singletons are included at the end.

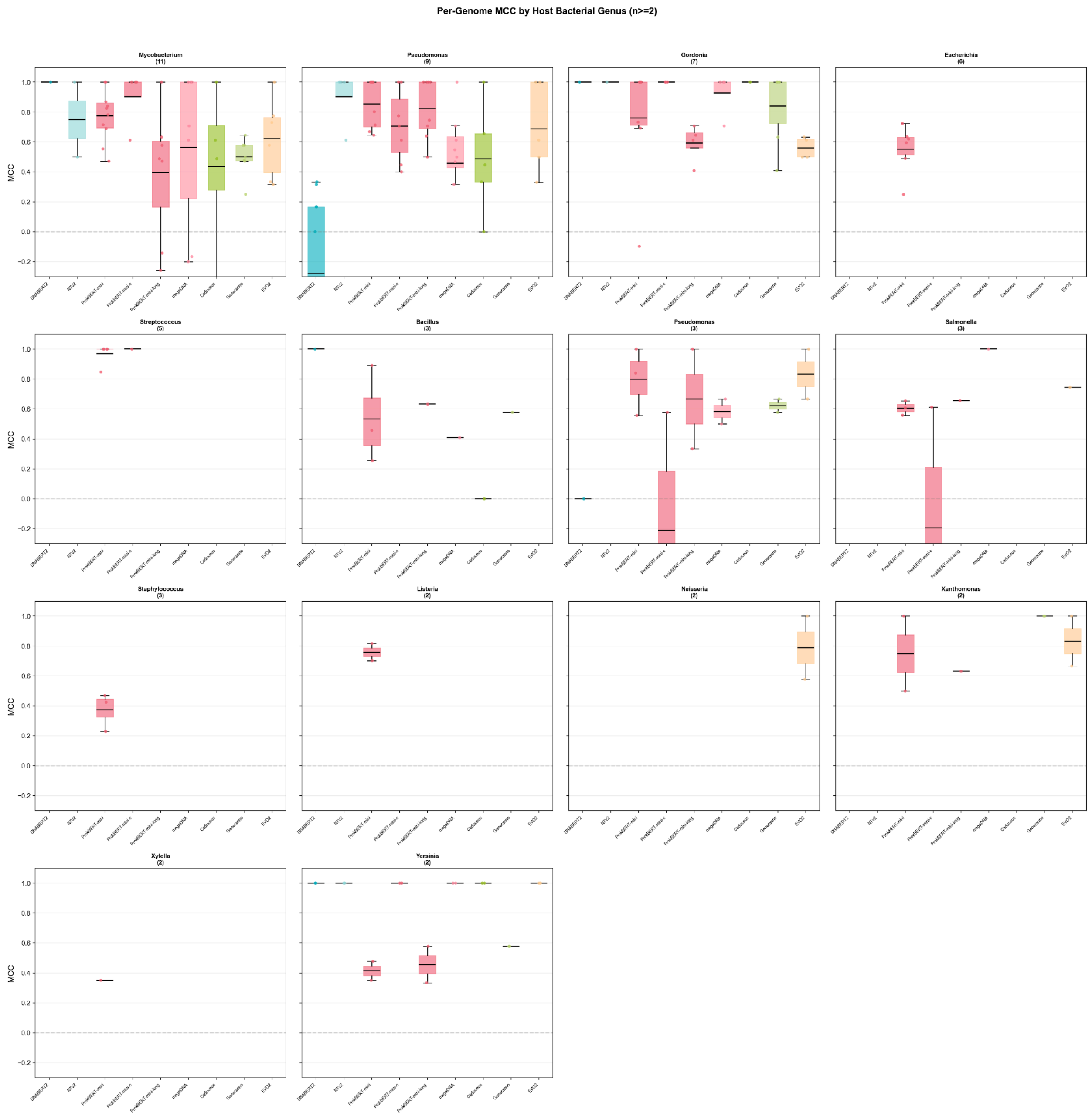

Figure S11: Model Performance Stratified by Bacterial Genome Taxonomy (Genus). Per-genome MCC of prophage detection by host bacterial genus (genera with n≥2 genomes). Groups are sorted by number of genomes (descending).

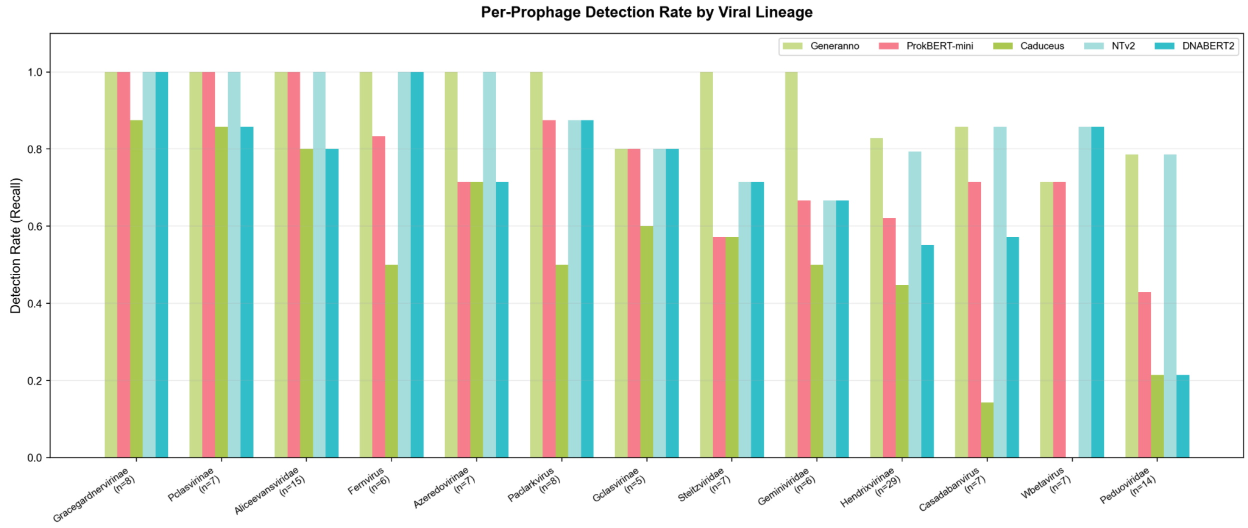

Figure S12: Model Performance Stratified by Viral Lineage. Per-prophage Jaccard index by viral lineage (lineages with n≥5 prophages). Lineage classifications were assigned by Pharokka. Higher Jaccard values indicate better spatial overlap between predicted and ground-truth prophage boundaries.

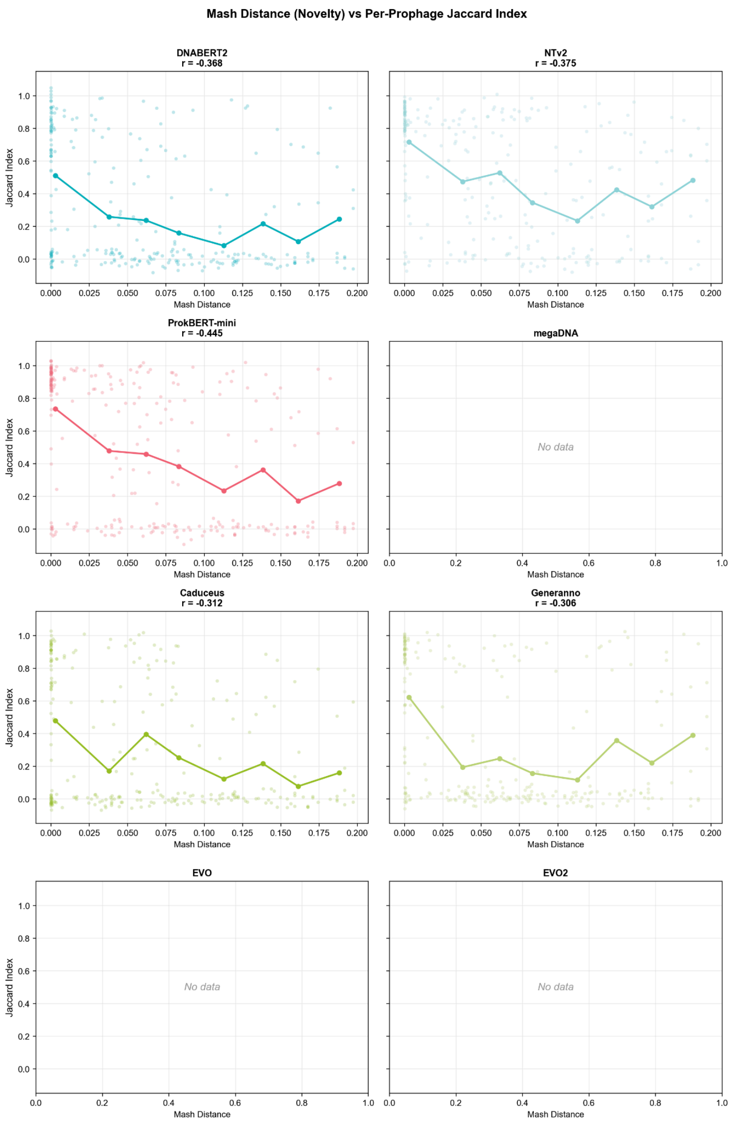

Figure S13: Model Performance compared to MASH distance to Nearest INPHARED Phage. Each panel shows the per-prophage Jaccard Index as a function of Mash distance to the nearest known phage in the INPHARED database, as determined by Pharokka. Individual prophages are shown as points, with lines connecting binned means to highlight trends. Pearson correlation coefficients (r) are reported for each model. All models with sufficient predictions show a negative correlation, indicating that prophages more distant from characterized phages are detected less accurately.

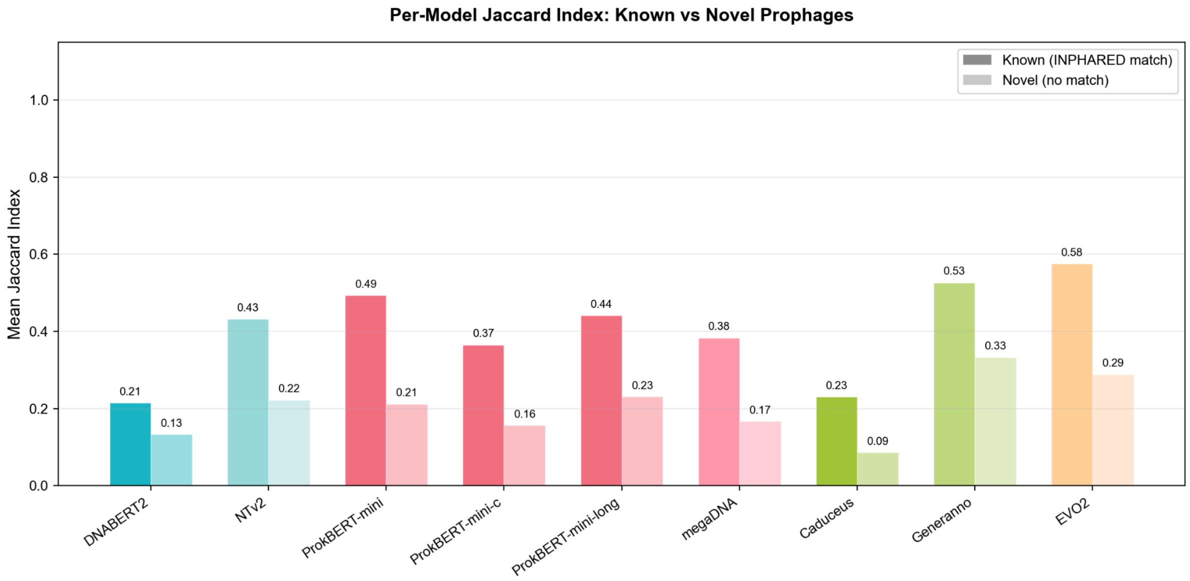

Figure S14: Per-model Mean Jaccard Index for Known vs. Novel Prophages. Per-model detection rate for known vs novel prophages. Mean Jaccard Index between predicted adn ground-truth prophage boundaries for prophages with a match in the INPHARED database (solid bars "Known") versus those with no match (hatched bars, "Novel"), Novelty was determined using Mash distance threshold of greater than 0.2 as computed by Pharokka. All models show reduced detection quality on novel prophages, with Generanno showing the smallest performance gap (0.36 vs 0.33)

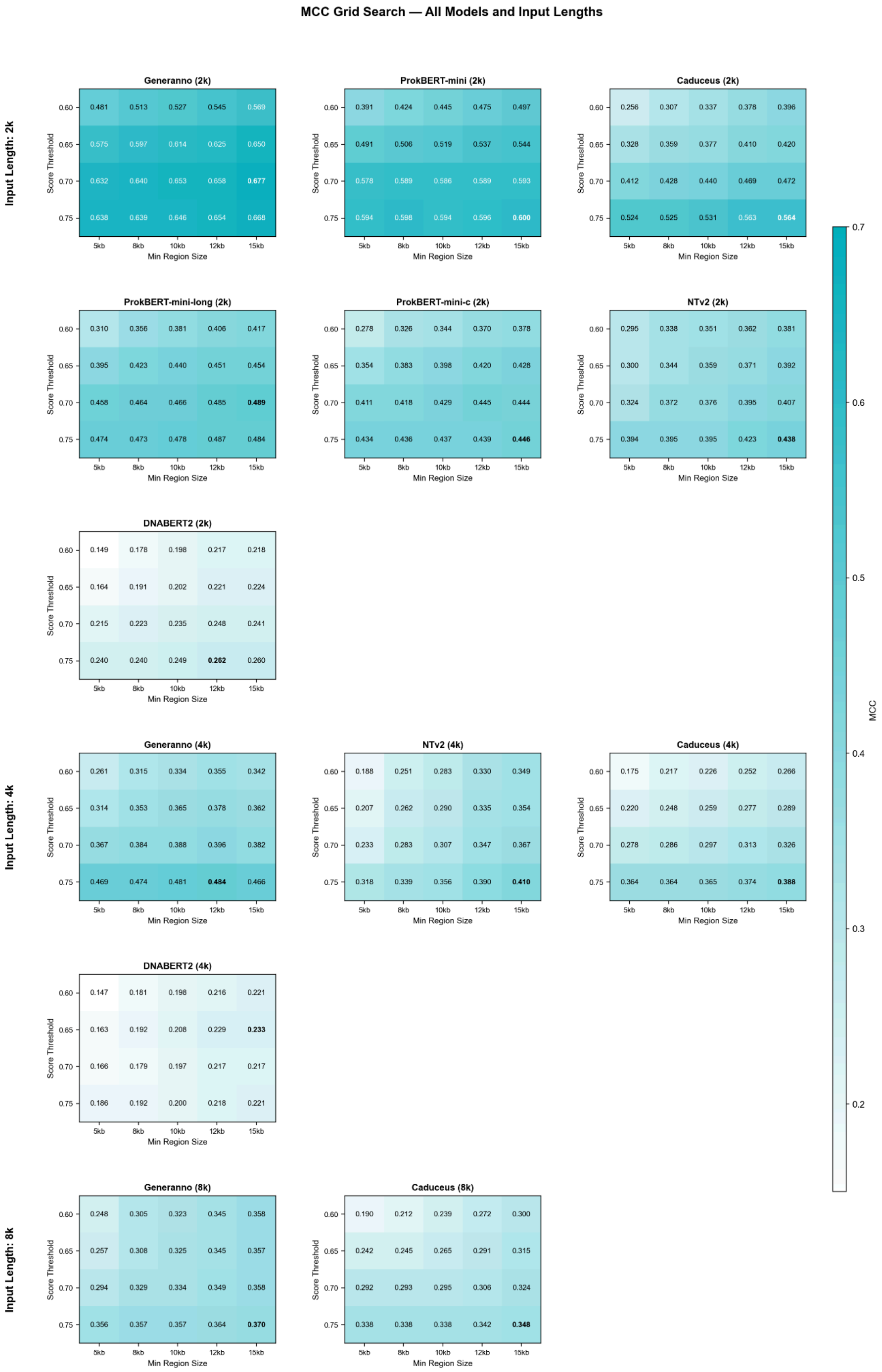

Figure S15: Hyperparameter Grid-Search for Prophage Signal Extraction Algorithm. Predicted regions were filtered by score threshold (y-axis), defined as the minimum average per-position prophage probability, and minimum region size (x-axis). Each cell reports the resulting genome-wide MCC for that parameter combination, with the best MCC per model shown in bold. Optimal filtering parameters for each model were used to report the genome wide phage prediction results.

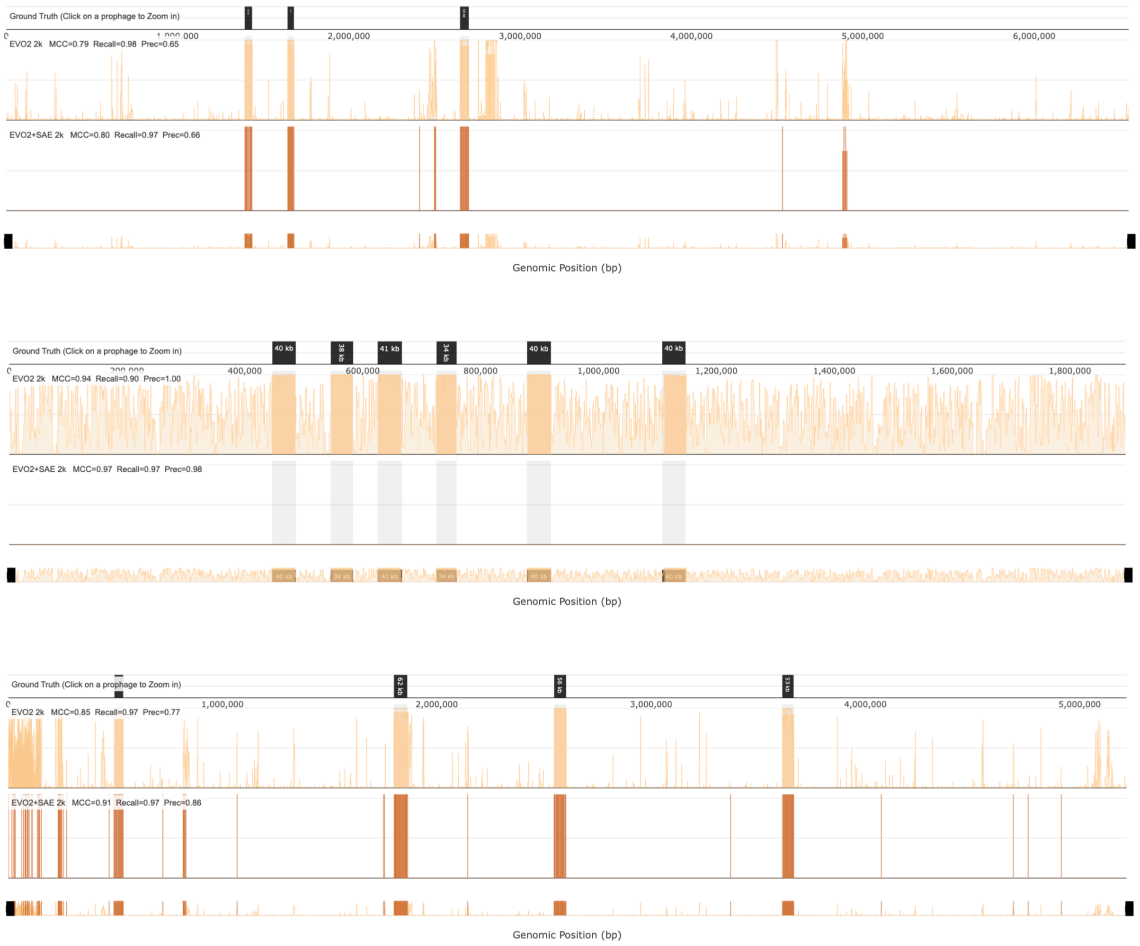

Figure S15: Genome-wide comparison of EVO2 and EVO2+SAE activations. Genome-wide tracks comparing raw EVO2 embedding-derived signal (top track in each panel) with activations from the sparse autoencoder applied to EVO2 (EVO2+SAE; middle track). The SAE-derived signal is markedly sparser and activates selectively, explaining its lower MCC score on the LAMBDA benchmark (0.636 vs. 0.680).

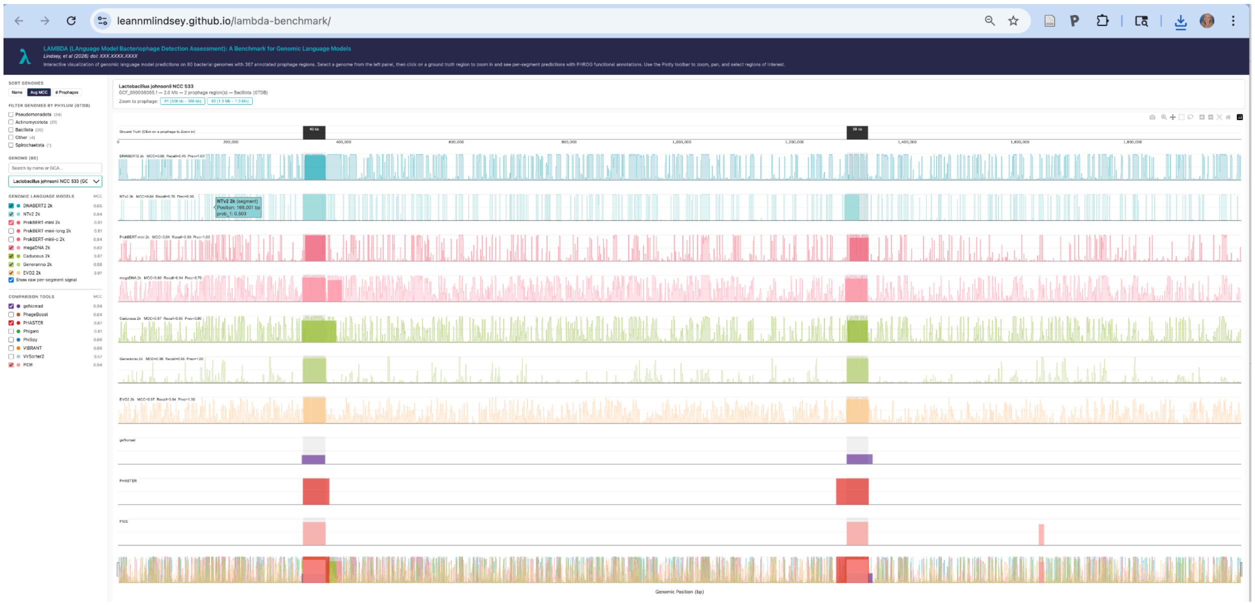

Figure S15: Website to Visualize the LAMBDA predictions. Screenshot of the LAMBDA interactive visualization tool, available at https://leannmlindsey.github.io/lambda-benchmark/. Users can explore genome-wide prophage predictions from each genomic language model across all 80 benchmark genomes, zoom into individual prophage regions, and view per-segment prediction scores alongside PHROG functional annotations.
